# Mechanical Properties and Degradation Behaviors of Zn-xMg Alloy Fine Wires for Biomedical Applications

**DOI:** 10.1155/2021/4831387

**Published:** 2021-12-24

**Authors:** Jing Bai, Yan Xu, Qizhou Fan, Ruihua Cao, Xingxing Zhou, Zhaojun Cheng, Qiangsheng Dong, Feng Xue

**Affiliations:** ^1^Jiangsu Key Laboratory for Advanced Metallic Materials, School of Materials Science and Engineering, Southeast University, Nanjing 211189, China; ^2^Institute of Medical Devices (Suzhou), Southeast University, Suzhou 215000, China; ^3^Jiangsu Key Laboratory of Advanced Structural Materials and Application Technology, School of Materials Science and Engineering, Nanjing Institute of Technology, Nanjing 211167, China

## Abstract

Zn and Zn-based alloys exhibit biosafety and biodegradation, considered as candidates for biomedical implants. Zn-0.02 wt.% Mg (Zn-0.02 Mg), Zn-0.05 wt.% Mg (Zn-0.05 Mg), and Zn-0.2 wt.% Mg (Zn-0.2 Mg) wires (*Φ* 0.3 mm) were prepared for precision biomedical devices in this work. With the addition of Mg in Zn-xMg alloys, the grain size decreased along with the occurrence of Mg_2_Zn_11_ at the grain boundaries. Hot extrusion, cold drawing, and annealing treatment were introduced to further refining the grain size. Besides, the hot extrusion and cold drawing improved the tensile strength of Zn-xMg alloys to 240-270 MPa while elongation also increased but remained under 10%. Annealing treatment could improve the elongation of Zn alloys to 12% -28%, but decrease the tensile strength. Furthermore, Zn-xMg wires displayed an increase in degradation rate with Mg addition. The findings might provide a potential possibility of Zn-xMg alloy wires for biomedical applications.

## 1. Introduction

Biomedical devices have been paid much attention since the living standard was improved during recent decades. Therefore, Mg-based, Zn-based, and Fe-based biodegradable metals (BMs) have become research hotspots owing to their acceptable mechanical properties, good biodegradability, and suitable biocompatibility for biomedical applications [1].

Fe-based alloys have higher mechanical properties similar to that of stainless-steel implants [2]. However, the degradation rates of Fe-based BMs were so slow in vivo that might hinder the bone healing process [3]. Besides, the ferromagnetism behaviors undermine the compatibility of magnetic resonance imaging (MRI) [4]. Besides, Mg-based alloys were investigated as BMs due to their excellent biocompatibility and suitable mechanical properties close to human bone [1, 2, 5, 6]. Mg ions play a positive role in promoting osteoblast proliferation and cell viability [7]. Nevertheless, an underlying problem is that a fast degradation rate destroys the mechanical integrity of implants during the healing of tissue [8, 9].

To explore suitable BMs with a moderate degradation rate and biomechanical compatibility, Zn and Zn alloys were investigated for biomedical applications [10–16]. Zn elements are also necessary microelements in the human body, which participate in life activities as major ingredients for vast macromolecules and enzymes in biomembrane formation [17, 18]. Besides, Zn has a standard electrode potential (vs standard hydrogen electrode) (-0.7618 V) between that of Mg (-2.372 V) and Fe (-0.447 V) [19], indicating a moderate degradation rate of Zn as BMs. Bowen et al. [20] studied pure Zn wires implanted in the artery of rats for 6 months and found that pure Zn wires had a proper degradation rate and favorable biocompatibility. However, the as-cast pure Zn has been reported limited elongation and tensile strength [21]. The drawbacks on undesirable plasticity and strength would be worthy of solving to expand clinical applications. The common metal materials are strengthened and plasticized through alloying and deformation combined with proper thermal treatment. Li et al. [22] reported that the mechanical properties of Zn alloys were improved by Mg, Ca, and Sr alloying and deformation process, resulting in that the ultimate tensile strength and elongation reached about 250 MPa and 7%, respectively. Zn-Mg alloys are widely studied among biodegradable Zn alloys [2]. Also, our research group prepared Zn-1.6% Mg alloy via multipass equal channel angular pressing and the optimal mechanical properties with the ultimate tensile strength of 423 MPa, the yield strength of 361 MPa, and the elongation of 5.2% [14]. In addition, Zn-Mg alloys have been reported good biocompatibility and osteogenesis in vivo and in vitro [22–24]. Therefore, Zn-Mg alloys are worthy of further investigation to explore wide biomedical applications.

Recently, BM wires have been paid much attention as temporary medical devices. Herein, BM sutures are potential biomedical applications owing to broadly accepted biodegradability, biocompatibility, and mechanical properties [25]. Our research group developed Mg alloy fine wire with a diameter of less than 0.4 mm [26]. Guo et al. [27] reported pure Zn wire for suture application. Zn-Mg alloys with a diameter of 0.6 mm have been developed for urinary tract surgeries [28]. BM fine wire with a diameter of less than 0.5 mm showed huge potentials in medical applications [26]. Zn alloy wires are appropriate for high strength sutures in hard tissue applications, which are required to have a long lifetime degradation of up to 2 years [25]. Furthermore, to explore precision medical implants with service performances, Zn-xMg (*x* = 0.02 wt.%, 0.05 wt.%, 0.2 wt.%) alloy fine wires (*Φ* 0.3 mm) were developed via hot extrusion, cold drawing, and annealing treatment. The microstructures and mechanical properties of different alloys through the whole process were analyzed and studied in detail. Besides, in *vitro* corrosion experiments were introduced to evaluate the corrosion properties of Zn-xMg alloy wires.

## 2. Materials and Methods

### 2.1. Material Preparation

The nutrient element Mg was selected as an alloying element for Zn-xMg alloys, including Zn-0.02 wt% Mg (Zn-0.02 Mg), Zn-0.05 wt% Mg (Zn-0.05 Mg), and Zn-0.2 wt% Mg (Zn-0.2 Mg). Pure Zn and Zn-xMg alloys were prepared by melting pure Zn (99.95%) and pure Mg (99.95%) in an iron crucible shielded by mixed gas (CO_2_ and SF_6_) using a resistance furnace. The molten metal was cast into a water-cooled cylindrical coppery mold (*Φ* 60 mm) to obtain the as-cast Zn alloy ingots.

Zn-xMg alloy ingots were annealed at 350°C for 24 h to acquire uniform microstructures and then hot extruded at 200-300°C and an extrusion rate of 25 : 1. Next, the hot-extruded Zn-xMg alloy wires with a diameter of *Φ* 3 mm were cold-drawn to *Φ* 0.3 mm by a self-made drawing machine at room temperature (RT) [27]. If broken during cold drawing, the Zn-xMg alloys were annealed at 200°C for 5 min. Finally, Zn alloy wires (*Φ* 0.3 mm) were annealed at different temperatures (100°C, 150°C, 200°C, 250°C, 300°C) for different times (10 min, 30 min) to enhance the plasticity.

### 2.2. Microstructure Characterization

The metallographic microstructure paralleled to the deforming direction was detected and collected using an optical microscope (Olympus, Shinjuku, Tokyo, Japan). Before observation, the samples were ground and polished to mirror surface and then etched by a mixed solution of CrO_3_ and Na_2_SO_4_ (10 g CrO_3_, 0.75 g Na_2_SO_4_, and 50 mL H_2_O) for 6-7 s at RT. The grain size was measured by the linear intercept technique [29]. Besides, the phases of the as-cast alloy were analyzed by an X-ray diffractometer (XRD, D8-Discover, German Bruker). The microstructures of cold-drawn alloy wires (*Φ* 0.3 mm) were characterized by synchrotron radiation X-ray diffractometer (SR-XRD) at BL14B1 station, SSRF (Shanghai, China). Moreover, the second phases in Zn-Mg alloys were identified by a scanning electron microscope (SEM, Sirion 200) equipped with an energy dispersive spectrometer (EDS).

### 2.3. Mechanical Properties

Mechanical properties, including ultimate tensile strength (UTS), yield strength (YS), and elongation to fracture (EL), were measured by a universal material test machine (CTM4503). The wires with a gauge size of 50 mm in the center and a clamping size of 20 mm at both ends were designed as tensile samples. The mechanical tests for each sample were repeated three times at a constant tensile speed of 2 mm/min at RT.

The microhardness of the Zn alloy wires was measured using a microhardness tester (FM-700, Future-Tech, Japan) with a loading force of 100 gf. The hardness values of Zn alloy wires were collected and analyzed based on ten testing points of each sample.

### 2.4. Corrosion Behaviors

Corrosion behavior was measured by electrochemical methods and immersion tests in Hank's solution at 37 ± 0.5°C. In these electrochemical measurements, an electrochemical station (CHI660E) was carried out to collect open circuit potential (OCP) and potentiodynamic polarization (PP) curves. A classical three-electrode cell was applied for electrochemical measurements, in which the working electrode was Zn alloy wires with a fixed exposed area in Hank's solution, the platinum electrode was set as a counter electrode, and a saturated calomel electrode was acted as a reference electrode. Prior to electrochemical measurements, Zn alloy wires were acid cleaned in 1% nitric acid solution to remove surface oxide films. The wires were inserted vertically in Hank's solution for electrochemical measurements. The immersion length of the wires was recorded to calculate the exposed surface area. OCP curves were measured for 2400 s in Hank's solution. PP curves were scanned from -1.6 V vs SCE to -0.6 V vs. SCE at a scanning rate of 1 mV/s. In immersion tests, the wires were cleaned in 200 g/L CrO_3_ solution at 80°C for corrosion product removal and dried before and after weighting. Corrosion rate (CR, mm/year) was calculated referring to Equation (1) [30], where *m*_0_ is the initial weight of specimens, *m*_1_ is the remaining weight of specimens after corrosion, *ρ* is the density of the alloy (7.138 g/cm^3^ for Zn-0.02 Mg, 7.137 g/cm^3^ for Zn-0.05 Mg, and 7.129 g/cm^3^ for Zn-0.2 Mg), and *t* is the corrosion time. The corrosion morphology and products after 7 d and 14 d immersion were observed by SEM equipped with EDS.

(1) $$\begin{array}{c} \text{CR}=\frac{3.65\times \left(m_{0}-m_{1}\right)}{\rho }. \end{array}$$

## 3. Results and Discussion

**Figure 1:**
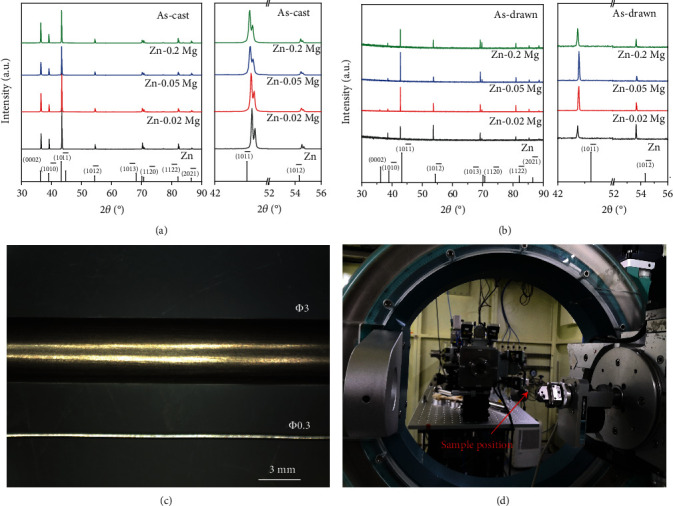
(a) XRD pattern of as-cast Zn-xMg alloys, (b) SR-XRD pattern of cold-drawn alloy wires, (c) optical morphology of alloy wires with 0.3 mm and 3 mm in diameter, and (d) synchronous radiator.

The chemical concentration of as-cast Zn and its alloys was detected by an optical emission spectrometer (OES), as exhibited in Table 1. The OES results are fitting well with the designed compositions, and a little fluctuation in chemical composition might be caused by melting loss. Figures 1(a) and 1(b) are XRD patterns of as-cast and as-drawn Zn alloys. The as-drawn Zn alloy wires were analyzed by a synchronous radiator (Figure 1(d)), in which SR-XRD was performed for phase identifying of precision devices including Zn alloy fine wires. The Zn phase (JCPDF No. 04-0831) was identified in the as-cast and as-drawn Zn alloys while Mg-Zn second phase could not be found owing to the little Mg addition. Zn alloy wires with the dimension of *Φ* 3 mm and *Φ* 0.3 mm are displayed in Figure 1(c), and the surfaces show metallic color after cold drawing. Moreover, the comparison between XRD and SR-XRD patterns indicated that the texture evolution is induced by extrusion and cold drawing. There is a drastic decrease in the relative intensity of (0002) and (101^−^0) planes, where the diffraction intensity of the (101^−^2) plane increases in Zn-xMg alloys. By comparison, the (101^−^2) plane of pure Zn is shifted to the strongest peak while Zn-xMg alloys still keep the (101^−^1) plane as the strongest peak, demonstrating that the Mg addition hinders the (101^−^2) plane. Also, the diffraction peaks of Zn and Zn-xMg alloys are shifted to a low diffraction angle, indicating the increase of d-spacing caused by deformation. As a consequence, Mg alloying in Zn alloys induces texture evolution during deformation.

**Figure 2:**
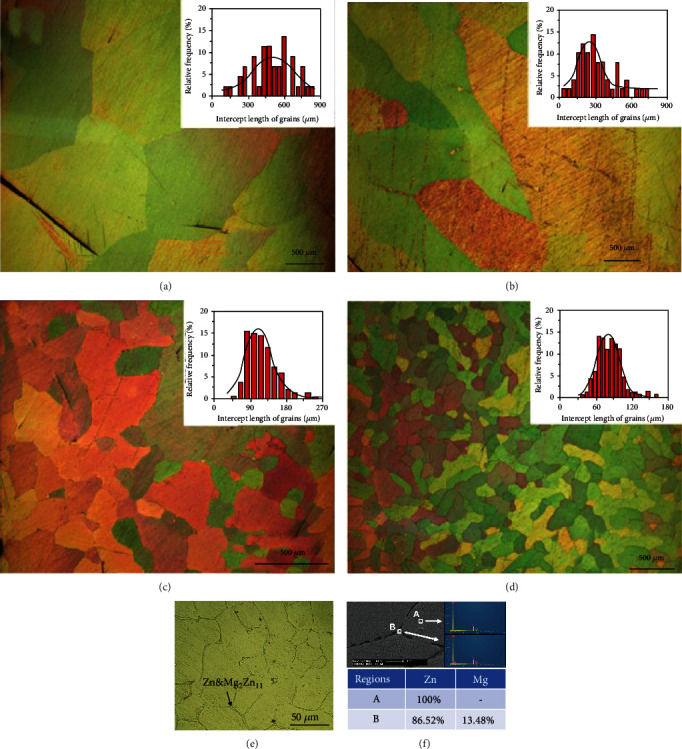
Microstructures of as-cast pure Zn (a), Zn-0.02 Mg (b), Zn-0.05 Mg (c) and Zn-0.2 Mg (d,e), and SEM and EDS for Zn-0.2 Mg (f).

Figure 2 exhibits the microstructures of as-cast Zn and Zn-xMg alloys. The average grain size of as-cast pure Zn is about 430 *μ*m (in Figure 2(a)). The grain sizes of Zn-xMg alloys decrease with Mg addition, which are about 250 *μ*m for Zn-0.02 Mg, 110 *μ*m for Zn-0.05 Mg, and 80 *μ*m for Zn-0.2 Mg (in Figure 2(b)–2(d)). According to the Zn-Mg binary phase diagram, the solubility of Mg in Zn at RT is 0.008%, and the maximum solubility is 0.16% at 364°C. Besides, the eutectic phases (*α* − Zn + Mg_2_Zn_11_) are found with the Mg addition exceeding 0.15% [14]. As shown in Figure 2(e), the eutectic phases are distributed uniformly in Zn-0.2 Mg alloys. Herein, the EDS results (in Figure 2(f)) indicated that the Mg_2_Zn_11_ phases are located in the grain boundary of Zn-0.2 Mg alloys.

**Figure 3:**
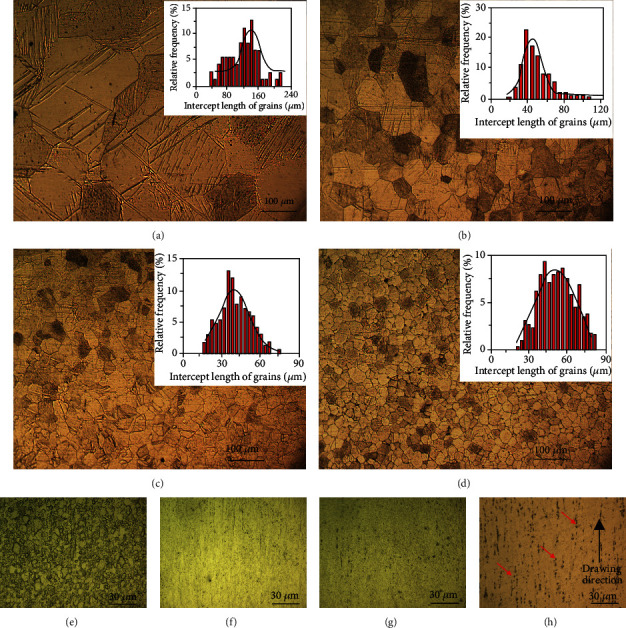
Microstructures of as-extruded Zn and Zn-xMg alloys: (a) Zn, (b) Zn-0.02 Mg, (c) Zn-0.05 Mg, and (d) Zn-0.2 Mg; microstructures of as-drawn Zn and Zn-xMg alloys: (e) Zn, (f) Zn-0.02 Mg, (g) Zn-0.05 Mg, and (h) Zn-0.2 Mg.

Figure 3 illustrates the microstructures of Zn and Zn-xMg alloys after hot extrusion and cold drawing. The as-extruded grains are equiaxed in shape and with smaller size. The grain size distribution graphs are analyzed as inserted in Figures 3(a)–3(d). With Mg addition from 0.02 wt% to 0.2 wt%, the grain size and relevant distribution range are shrunk obviously. The average grain sizes of the as-extruded Zn alloys are about 130 *μ*m for pure Zn, 60 *μ*m for Zn-0.02 Mg, 40 *μ*m for Zn-0.05 Mg, and 20 *μ*m for Zn-0.2 Mg, respectively. Besides, after 54 passes cold drawing without annealing, the material starts dynamic recrystallization so that the fine and equiaxed grains result see in Figure 3(e), while the original equiaxed grains of as-extruded Zn-xMg are transformed into fibrous structures along the drawing direction. As indicated by red arrows in Figure 3(h), the intermediate phases are dispersed along the drawing direction in Zn-0.2 Mg alloys.

**Figure 4:**
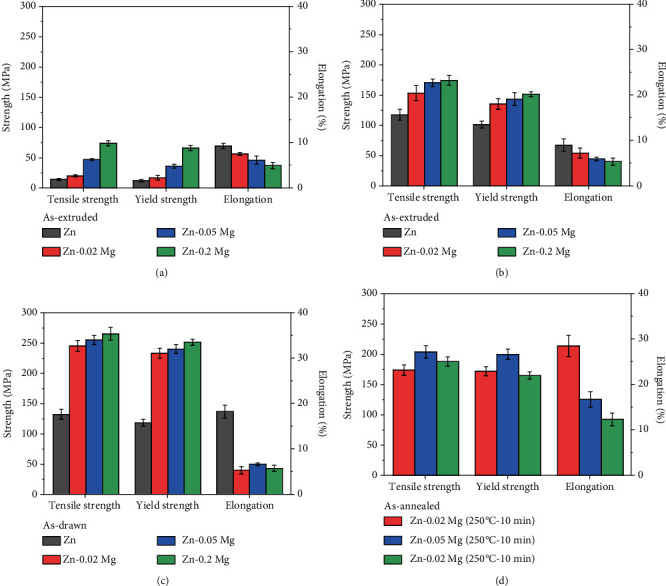
Mechanical properties of pure Zn and Zn-xMg alloys in different states: (a) as-cast, (b) as-extrude, (c) as-drawn, and (d) as-annealed.

Figure 4 summarizes the mechanical properties of Zn and Zn-xMg alloys. In Figure 4(a), with Mg addition, the as-cast Zn and Zn-xMg alloys appear extremely brittle with reduced ELs and improved mechanical strength including UTS and YS. The as-cast Zn has the lowest UTS of 14 MPa while UTS of Zn-0.2 Mg reaches the maximum of 74 MPa. The strengthening effect results from grain refinement and the formation of second phases. Figures 4(b) and 4(c) show the mechanical properties of pure Zn and Zn-xMg alloys after the deformation process. After hot extrusion, both the grain refinement and dispersed Mg_2_Zn_11_ in Zn-xMg alloys contribute to an enhancement in mechanical properties, which UTS reaches 117 ± 8.9 MPa for pure Zn and 174 ± 7.7 MPa for Zn-0.2 Mg. The as-extruded Zn alloys have a grain size > 20 *μ*m, in which grain boundary sliding can be activated. Thus, the as-extruded Zn alloys have limited ELs below 10%. As shown in Figure 4(c), there is an increasing trend in UTSs and YSs of as-drawn Zn and Zn alloys. UTSs increase with Mg addition: the as-drawn Zn wire has UTS of 130 MPa, and Zn-0.2 Mg wire shows an UTS of 270 MPa. By contrast, it is found that there are no changes in ELs of Zn-xMg alloys but an obvious increase in EL of pure Zn to 18.2%, which is attributed to the dynamic recrystallization at RT. Liu et al. reported the extraordinary plasticity of as-cast pure zinc is attributed to dynamic recrystallization [31]. However, the dynamic recrystallization might be restrained by the solid solution and second phase; so, the equiaxed crystals cannot be found in the as-drawn Zn-xMg alloys. Furthermore, the as-drawn Zn-xMg alloy wires are heat-treated at 250°C for 10 min, and the as-annealed Zn-xMg alloy wires exhibit comprehensive mechanical properties with combined strength and plasticity.

**Figure 5:**
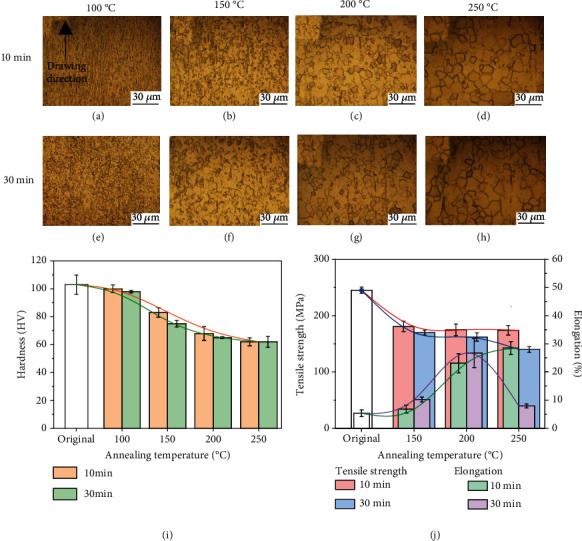
Microstructures and mechanical properties of Zn-0.02 Mg wire annealed under different conditions: (a) 100°C-10 min, (b) 150°C-10 min, (c) 200°C-10 min, (d) 250°C-10 min, (e) 100°C-30 min, (f) 150°C-30 min, (g) 200°C-30 min, (h) 250°C-30 min, (i) microhardness, and (j) tensile strength and elongation.

Moreover, the work also investigated the effect of annealing on microstructure and mechanical properties.  Figure 5 exhibits the microstructures and mechanical properties of as-annealed Zn-0.02 Mg. Both, raising treatment temperature and prolonging time, coarsen microstructures regulate mechanical properties. In the recovery process at 100°C, the hardness seems to give no obvious sign of slowing down along with a slight increase in grain size. When the annealing temperature is above 100°C, the microhardness decreases sharply with the weakness of the inherited structure and occurrence of equiaxial grains, indicating the recrystallization process. The completed recrystallization might be performed at temperature above 200°C, in which the microhardness tends to be stable.  Figure 5(j) shows the UTS and EL results of the as-annealed Zn-0.02 Mg. As the annealing temperature rises, UTS decreases in opposite to the increase of EL. Meanwhile, with annealing prolonged to 30 min, it is obvious that UTS decrease and EL increases. However, annealed at 250°C for 30 min, the excessive grain coarsening also makes damage to the improvement of EL. As mentioned above, the optimized parameter for annealing is at 250°C for 10 min, and the as-annealed Zn-0.02 Mg wire has an UTS of 172 MPa and EL of 28.5%. The short-time annealing at high temperature might complete recrystallization and retard excessive grain coarsening, leading to an increase in EL of the annealed Zn alloy wires.

**Figure 6:**
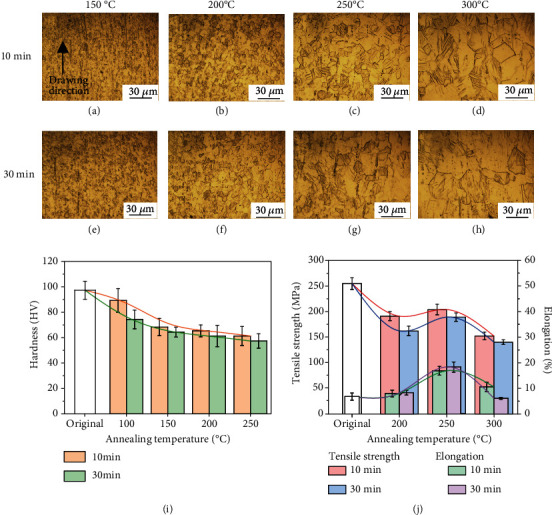
Microstructures and mechanical properties of Zn-0.05 Mg wire annealed in different parameters: (a) 150°C-10 min, (b) 200°C-10 min, (c) 250°C-10 min, (d) 300°C-10 min, (e) 150°C-30 min, (f) 200°C-30 min, (g) 250°C-30 min, (h) 300°C-30 min, (i) microhardness, and (j) tensile strength and elongation.

**Figure 7:**
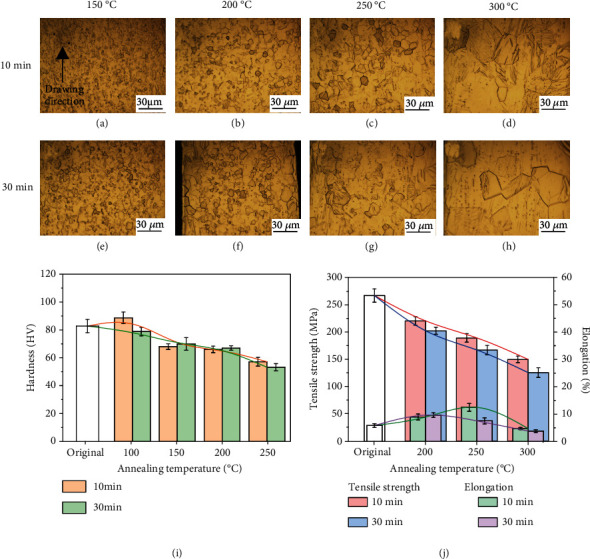
Microstructures and mechanical properties of Zn-0.2 Mg wire annealed in different parameters: (a) 150°C-10 min, (b) 200°C-10 min, (c) 250°C-10 min, (d) 300°C-10 min, (e) 150°C-30 min, (f) 200°C-30 min, (g) 250°C-30 min, (h) 300°C-30 min, (i) microhardness, and (j) tensile strength and elongation.

Figures 6(a)–6(h) and 7(a)–7(h) display the microstructures of the annealed Zn-0.05 Mg and Zn-0.2 Mg alloy wires. An increase in Mg hinders the reversion and recrystallization, and a higher temperature is applied for annealing. The microstructure and microhardness evolution of Zn-0.05 Mg and Zn-0.2 Mg alloys are similar to that of the Zn-0.02 Mg alloy. The microstructure evolution process is composed of recovery, recrystallization, and coarsening stages. Figures 6(i), 6(j), 7(i), and 7(j) display the hardness and tensile test results. By comparison, the annealing temperature has more significant effects on microstructure and mechanical properties than the treatment time. In conclusion, Zn-xMg alloy wires have a desirable microstructure and optimized mechanical properties after annealed at 250°C for 10 min. Annealed at the optimized parameter, the Zn-0.05 Mg alloy wire has an UTS of 204 MPa and EL of 16.7% while the Zn-0.2 Mg alloy wire has an UTS of 165 MPa and EL of 12.3%. Herein, the plasticity of Zn-0.2 Mg alloy wire was improved with limits owing to more intermediate phases.

**Figure 8:**
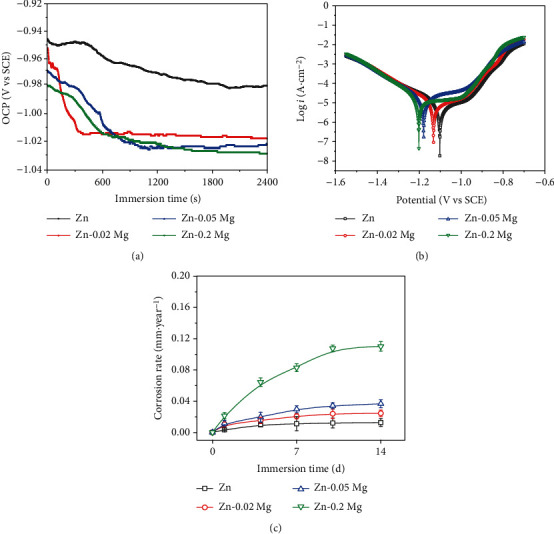
Corrosion measurements: (a) OCP curves, (b) PP curve, and (c) corrosion rate measured by weight loss.

Figure 8 exhibits the electrochemical results of cold-drawn Zn and annealed Zn-xMg alloy wires, and Table 2 lists the corrosion rate (CR) calculated by electrochemical methods and weight loss measurements. After the initial immersion in Hank's solution, OCP values (in Figure 8(a)) are stable to about -0.98 V (vs SCE) for pure Zn, -1.02 V (vs SCE) for Zn-0.02 Mg, -1.02 V (vs SCE) for Zn-0.05 Mg, and -1.03 V (vs SCE) for Zn-0.2 Mg, respectively. Mg addition for Zn alloys increases the tendency towards corrosion. In PP curves, Zn-xMg alloy wires exhibit larger corrosion current density (*i*_corr_) with Mg addition. The *i*_corr_ is a major evaluation criterion of corrosion rate [32]. The corrosion rate is calculated from the electrochemical results, as shown in Figure 8(b). Zn-0.05 Mg and Zn-0.2 Mg alloy wires display rapid degradation rates. Moreover, the long-time immersion tests directly reflect the degradation behavior of the Zn alloys after implantation.  Figure 8(c) shows the corrosion rate curves based on weight loss tests. Zn-0.2 Mg alloy wire is degraded at a relatively higher rate while pure Zn wire has the lowest degradation rate. The mentioned above reflects that Mg alloying is conducive to improving the biodegradability of Zn alloys for biomedical applications.

**Figure 9:**
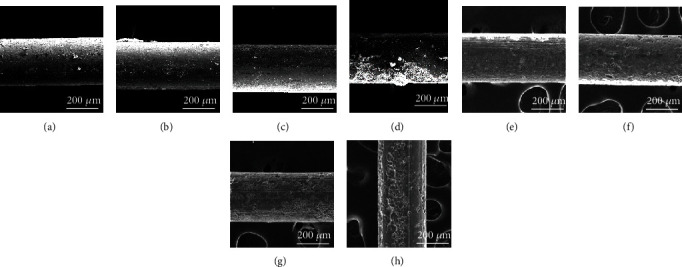
Morphologies of Zn-xMg wires immersed for 7 days: (a) pure Zn wire, (b) Zn-0.02 Mg wire, (c) Zn-0.05 Mgwire, and (d) Zn-0.2 Mg wire. Corrosion morphologies of Zn-xMg wires immersed for 14 days: (e) pure Zn wire, (f) Zn-0.02 Mg wire, (g) Zn-0.05 Mgwire, and (h) Zn-0.2 Mgwire.

**Figure 10:**
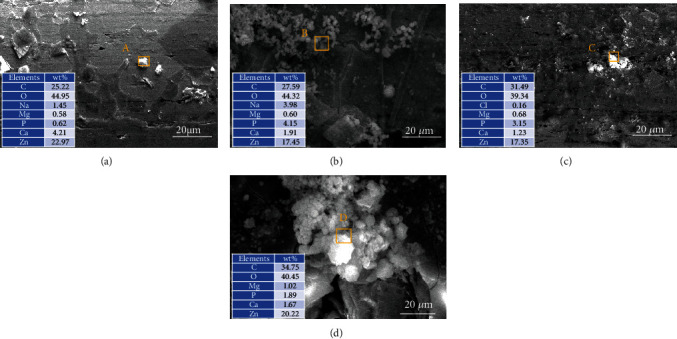
Morphologies and EDS of corrosion products on the surface of Zn-xMg wires immersed for 7 days: (a) pure Zn wire, (b) Zn-0.02 Mg wire, (c) Zn-0.05 Mg wire, and (d) Zn-0.2 Mg wire.

In order to further evaluate the degradation behavior, the surface morphologies of Zn and Zn-xMg alloy wires after immersion are exhibited in Figures 9(a)–9(h). Few corrosion pits are found in Zn wires after 7 d and 14 d immersion. Zn-xMg alloy wires are seriously corroded with Mg addition. Besides, the corrosion product morphologies are shown in Figures 10(a)–10(d). Zn-0.2 Mg alloy wire is covered by a mass of corrosion products, which is attributed to the rapid degradation of Mg-rich second phases. Besides, the rapid formation of corrosion products might temporarily slow down the corrosion rate during PP measurements after the initial immersion of 30 min. Thus, for Zn-0.2 Mg alloy wire, the corrosion rate calculated by PP seems to mismatch with that one measured by weight loss. Based on EDS analysis, the corrosion products of Zn alloys in Hank's solution are composed of Zn, O, and C elements as well as a few Mg, Ca, and P elements, indicating that the corrosion product is composed of complex Zn mineral phases [33], which might be ZnO, Zn_5_(CO_3_)_2_(OH)_6_, Zn_3_(PO_4_)_2_·4H_2_O, and Ca-P compounds [34]. With immersion time prolonging to 14 d, many deeper and more intensive corrosion pits occur on Zn-xMg alloys, especially Zn-0.2 Mg alloys, as shown in Figures 9(e)–9(h). These results are corresponding to that ones in Figure 8.

## 4. Conclusions

In this study, Zn-xMg (*x* = 0.02, 0.05, 0.2 wt%) alloy wires were prepared and investigated for biomedical applications. For the as-cast Zn and Zn-xMg alloys, Mg alloying led to an obvious decrease in average grain size and improves the tensile properties. Hot extrusion and cold drawing can substantially enhance the UTSs of Zn-xMg alloy and increase the ELs (5-8%) to a limited degree. Deformation texture was formed during the production, especially for Zn alloys with Mg alloying. Annealing treatment refined the structures of Zn-xMg alloy which further improved the plasticity but slightly decreased strength. After the optimized annealing process, Zn-0.05 Mg had the desirable comprehensive mechanical properties with UTS and EL reaching 204 MPa and 16.7%, respectively. Moreover, based on electrochemical measurements and immersion tests, Mg alloying increased the degradation rates of Zn alloy wires in Hank's solution. The research explored Zn-xMg alloy fine wires for biomedical applications and investigated the effect of the preparation process on service performances of Zn-xMg alloy fine wires.

## Figures and Tables

**Table 1:** Chemical composition analyzed by OES.

Materials	Composition (wt.%)								
	Mg	Al	Cu	Pb	Sn	Fe	Mn	Ni	Zn
Zn	0.0012	0.0120	0.0013	0.0092	0.0064	0.0075	0.0010	0.0009	Balance
Zn-0.02 mg	0.0223	0.0018	0.0014	0.0100	0.0110	0.0070	0.0025	0.0017	Balance
Zn-0.05 mg	0.0488	0.0013	0.0013	0.0105	0.0127	0.0083	0.0027	0.0020	Balance
Zn-0.2 mg	0.193	0.0013	0.0014	0.0103	0.0142	0.0090	0.0043	0.0021	Balance

**Table 2:** Corrosion rate calculated by electrochemical methods and weight loss measurements.

Alloy	Methods	Zn	Zn-0.02 mg	Zn-0.05 mg	Zn-0.2 mg
CR_1_ (mm/year)	Electrochemical methods	0.019 ± 0.002	0.029 ± 0.003	0.040 ± 0.005	0.036 ± 0.010
CR_2_ (mm/year)	Weight loss measurements	0.010 ± 0.001	0.020 ± 0.001	0.030 ± 0.002	0.104 ± 0.002

## Data Availability

The data used to support findings of this study are included within the article.
